# Internal jugular vein valve insufficiency is not increased in migraine: an ultrasound study in migraine patients and control participants

**DOI:** 10.1186/1129-2377-14-78

**Published:** 2013-09-23

**Authors:** Izabela Domitrz, Grzegorz Styczynski, Justyna Wilczko, Lucja Gadomska, Beata Parzuchowska, Wojciech Domitrz, Anna Kaminska

**Affiliations:** 1 Department of Neurology, Medical University of Warsaw, 1a Banacha Street, 02-097 Warsaw, Poland; 2 Department of Internal Medicine, Hypertension and Angiology, Medical University of Warsaw, Warsaw, Poland; 3 Faculty of Mathematics and Information Science, Warsaw University of Technology, Warsaw, Poland

**Keywords:** Migraine, Jugular vein valve incompetence, Headache

## Abstract

**Background:**

Migraine is a common neurological disorder of unclear pathogenesis. Recently incompetence of internal jugular vein valve (IJVVI) was found to be associated with some neurological conditions of unknown etiology such as benign cough headache, primary exertional headache or transient global amnesia. Common vascular mechanism linking transiently increased cerebral venous pressure with the above mentioned conditions was then postulated. Therefore we decided to investigate whether IJVVI may be associated with migraine.

**Aim and methods:**

The aim of our study was to evaluate the occurrence of IJVVI and retrograde flow duration in 70 (56 females) migraine patients by color Doppler ultrasound during Valsalva maneuver.

We assessed internal jugular vein valve in 44 patients with migraine without aura (39 female); mean age 37 ± 9 yrs. and in 26 patients with migraine with typical aura (17 female); mean age 34 ± 9 yrs. Age- and sex-matched control group consisted of 42 healthy persons (33 female); mean age 32 ± 1 yrs.

**Results:**

Frequency of the internal jugular vein valve insufficiency was similar in patients with migraine and in the healthy subjects (51% v. 40%, p = 0.26). Also mean values of retrograde flow duration were similar in both groups (2.4 ± 0.8 sec in migraine group and 2.2 ± 1.2 sec in controls, p = 0.14).

**Conclusion:**

The results of our study show no evidence for an increased prevalence of IJVVI in migraine patients.

## Background

Migraine is a common neurological disorder of uncertain pathogenesis characterized by a distinctive headache and in some patients transient focal neurological symptoms of its aura. Numerous hypotheses concerning the mechanism of a migraine attack include disturbances of the blood flow in the brain vessels resulting from pathological vasomotor regulation, probably associated with neuronal processes. In some neurological disorders of unknown pathogenesis the role of internal jugular vein valve incompetence (IJVVI) was recently discussed [1-5]. Internal jugular vein valve is the only venous valve between heart and brain and is thought to prevent transmission of increased venous chest pressure during Valsalva – like maneuvers (cough, straining, defecation, etc.) to cerebral venous system. Thus IJVVI may lead to a transient increase in the cerebral venous pressure and subsequently in the intracranial pressure. It is also possible that in patients with severe IJVVI some amount of blood from splanchnic circulation that mixes in the right atrium may theoretically be transported backwards into the internal jugular vein. There were some observations demonstrating aggravation of migraine during cerebral venous congestion. Two studies [6,7] demonstrated migraine aggravation during performing Queckenstedt’s test. Chou et al. [6] performed compression over bilateral internal jugular veins in 33 migraine patients. Doepp et al. [7] studied 25 patients with migraine without aura. Both of these studies suggested a role of cerebral venous congestion in migraine attack. However the another study, published in 1998, contradicted the role of the cephalic venous system in the migraine mechanism [8]. Chuang et al. [1] reported internal jugular or vertebral venous regurgitation in benign cough headache. Doepp et al. [2] reported incompetence of the internal jugular valve in patients with primary exertional headache. Some authors found a significantly increased frequency of IJVVI in the Transient Global Amnesia (TGA) patients [3-5]. IJVVI was also postulated to play role in the mechanism of cough syncope [9,10]. Internal jugular vein valve abnormalities were rarely assessed in primary headache patients. There is only one recent paper showing no association between migraine and IJVVI [11]. We decided to perform our study because of the unknown pathophysiology of migraine and the results of other studies suggesting the role of IJVVI in the pathophysiology of TGA [3-5] or cough [1] and exertional [2] headaches. Therefore, we aimed to evaluate possible internal jugular vein valve abnormalities in migraine patients as a potential reason for migraine attacks, and to discuss possible connections between these illnesses and migraine without or with aura.

## Methods

### Patients and methods

The study group consisted of 70 consecutive patients (mean age 36 ± 10 years) of The Outpatient Headache Clinic, with migraine without (MO) and with typical aura (MA) diagnosed according to the International Headache Society (IHS) criteria, 3^rd^ edition [12]. The diagnosis was made by an experienced neurologist working for more than 15 years in the Outpatient Headache Clinic. Duration of the disease ranged between 1 and 33 years. Frequency of migraine attacks ranged between 1 attack/ 1 week to 1 attack/ 6 months (mean frequency 1 attack/ 1 month). Attacks of migraine were bilateral in all patients, but in 13 migraine patients (8 MO, 5 MA) considerable side dominance was present.

**Figure 1:**
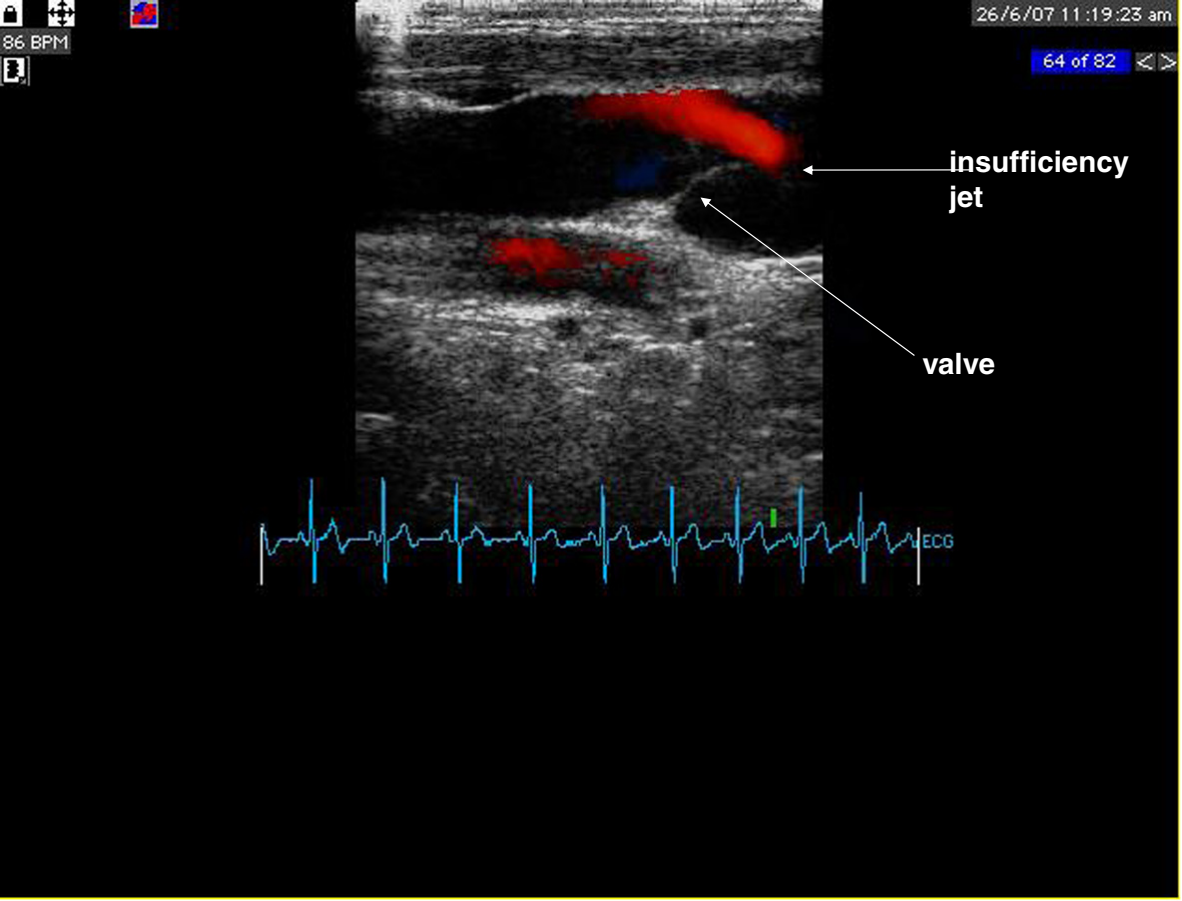
**Ultrasound evaluation of internal jugular valve incompetence in colour Doppler mode.** Insufficiency jet originating from the valve (red colour) is indicated by an arrow.

Demographic characteristics of the study population is presented in Table 1. In all migraine patients we performed color duplex ultrasound of internal jugular veins during Valsalva maneuver (VM) [13]. Ultrasound examinations were performed between migraine attacks. IJVVI evaluation was performed bilaterally, with the use of a high frequency (10 MHz) vascular probe connected to the ultrasound unit (Vingmed, System Five, GE) with commercial carotid artery presets. Study was performed in a supine position, after at least 10 minutes of rest. Before evaluation, (with the use of mouthpiece mounted on a flexible tube connected to manometer) patients were trained to perform Valsalva maneuver with expiratory effort equivalent to 50 mmHg during 5 seconds. Initially distal part of the internal jugular vein close to the valve was visualized in 2 dimensional mode, then with color mode at rest and during Valsalva maneuver. Assessment of IJVVI was based on the presence of color jet extending proximally from the valve (Figure 1). At the end of the Valsalva maneuver the image was frozen and the duration of the insufficiency jet was measured by reviewing backward the cineloop. Diagnosis of IJVVI was made when duration of the jet exceed 1.2 s. According to Nedelmann et al. study [13] shorter duration of the jet may reflect normal phenomenon associated with closure of the valve, and 1.23 s was the shortest duration of backward flow found in insufficient valves. All ultrasound studies were performed by single physician experienced in echocardiography and vascular ultrasound who was blinded to the patients’ diagnosis.

**Table 1:** Demographic characteristics of the study population

**Patients**	**Sex male/female (male)**	**Mean age ± SD years**
**M**	14/56^*^	36 ± 10^*^
n = 70	(20%)	(17 to 61 yrs.)
**MO**	5/39^*^	37 ± 9^*^
n = 44	(11%)	(19 to 61 yrs.)
**MA**	9/17^*^	34 ± 9^*^
n = 26	(35%)	(17 to 58 yrs.)
**Control group**	9/33^*^	32 ± 11^*^
n = 42	(21,5%)	(20 to 53 yrs.)
**P**-**values**	Chi square:	ANOVA:
	χ ^2^ = 4.04	F (2,109) = 2.562
	p = 0.13	p = 0.08

*M*, all patients with migraine; *MO*, patients with migraine without aura;

*MA*, patients with migraine with aura;

^*^no difference between the study groups p > *0*.*05*.

We analyzed the possible relationship between the side of IJVVI and the dominant side of the pain. Our data on regurgitation of internal jugular vein valve frequency were compared to the results of the age- and sex- matched control group (Table 1) which consisted of 42 healthy persons without a history of any headaches and cardiovascular problems. Controls were recruited from our students, hospital staff and our families. All individuals in the control group negated any headaches. They were screened by a general practitioner and then verified by an experienced neurologist.

The proposal for the study was presented to the ethical committee of Warsaw Medical University and was approved by this committee. Written informed consent was provided by all participants.

### Statistical analysis

Statistical analysis of the data was performed using Statistica 10. Statistical calculations were carried out using the test t-Student’s, Chi square and normal distribution test with p < 0.05 used as a cut-off value. The t-test was used for preliminary comparison of the mean age in the migraine group versus the control group. Chi square test was used to compare the sex between study groups. For normalcy we used Kolmogorov-Smirnov and Shapiro-Wilk tests. The multivariable analysis of variance (MANOVA) were calculated using the left and right duration of insufficiency flow in all study groups as the dependent variables. For the MANOVA group (MO, MA, controls) was between subjects factor. P-values < 0.05 were considered significant. These results were confirmed with Wilksa’s, Pillai’s, Roy’s and Lawley-Hotelling’s tests.

## Results

The results of IJVVI frequency are presented in Table 2. No significant group differences in the frequency of IJVVI and duration of the insufficiency jet were found in our study.

**Table 2:** Prevalence of internal jugular vein valve insufficiency

**Study groups**	**Bilateral or unilateral**	**Bilateral**	**Left-sided**	**Right-sided**
**M** n = 70	36 (51%)^*^	8 (11%)^*^	17 (24%)^*^	27 (39%)^*^
**MO** n = 44	23 (52%)^*^	7 (16%)^*^	13 (30%)^*^	17 (39%)^*^
**MA** n = 26	13 (50%)^*^	1 (4%)^*^	4 (15%)^*^	10 (38%)^*^
**Control group** n = 42	17 (40%)^*^	5 (12%)^*^	12 (28%)^*^	10 (24%)^*^

*M*, all patients with migraine; *MO*, patients with migraine without aura;

*MA*, patients with migraine with aura;

^*^no difference between the study groups p > *0*.*05*.

The presence of any unilateral (right or left side) or bilateral (right and left sides in the same patients) IJVVI was found in 51% of all migraine patients (compared to 40% of the control group; p = 0.26) and in 52% patients with migraine without aura (compared to 40% of the control group; p = 0.26). The presence of IJVVI was found in 50% of the patients with migraine with aura (MA), with no statistical significance compared to 40% of the control group (p = 0.51). Bilateral IJVVI was found in 11% of migraine patients without differences between the study groups (16% of MO, 4% of MA) and 16% of the control group (p = 0.85, p = 0.59, p = 0.25).

In 13 patients with side dominance of the pain during migraine attacks 2 patients had IJVVI on the side of the pain and 11 on the opposite side. MANOVA showed that there was no difference in the presence of IJVVI in three groups of different sides of the pain (both, left, right) F(4,126) = 0.32 (Wilks’ test), p = 0.86. ANOVA for the right side F(2,64) = 0.40, p = 0.67 and the left side F(2,64) = 0.34, p = 0.71 proved to be the same.

MANOVA did not show significant differences in the duration of the flow for unilateral or bilateral IJVVI between patients separately with MO or patients with MA and controls (F(4,216) = 1.76; p = 0.14). The same results were shown in Wilksa’s, Pillai’s, Roy’s and Lawley-Hotelling’s tests with p > 0.05. ANOVA for the left (F(2,109) = 2.101; p = 0.13), right IJVVI (F(2,109) = 1.424; p = 0,25) or bilateral IJVVI (F(2,10) = 0.542; p = 0.59) also did not show significant differences. The results of mean values of flow duration are presented in Table 3.

**Table 3:** **Internal jugular valve vein incompetence**: **mean values of flow duration** ± **SD in seconds**

**Study groups**	**Bilateral or unilateral IJVVI**	**Bilateral IJVVI**
**M** n = 70	2.4 ± 0.8^*^	4.8 ± 1.65^*^
**MO** n = 44	2.6 ± 0.9^*^	5.1 ± 1.6^*^
**MA** n = 26	1.9 ± 0.3^*^	3^*^
**Control group** n = 42	2.2 ± 1^*^	5.1 ± 1.2^*^

*M* all patients with migraine; *MO* patients with migraine without aura;

*MA* patients with migraine with aura;

^*^no difference between the study groups p > *0*.*05*.

## Discussion and conclusion

The results of our study show no relationship between migraine and IJVVI. We did not find increased frequency of IJVVI in migraine patients with or without aura comparing with the control group. Also retrograde flow duration results were similar and not significantly different in migraine patients and controls. Our study is one of the first studies which assessed the prevalence of IJVVI in migraine. It confirms negative results recently published by Rabe et al. [11]. The authors investigated IJVVI in 36 patients with aura, 50 without aura and 43 controls and they concluded that the prevalence of IJVVI is not increased in persons with migraine. The number of patients of our study groups were similar to Rabe’s et.al. [11] however frequency of IJVVI in our patients and control group were significantly higher and comparable to other reports in the literature [13,14]. Unilateral or bilateral jugular vein valve incompetence ranges from 29% in young adult population to 33-45% in healthy elderly subjects [13,14]. In our control group we found IJVVI in 40%, in accordance with published reports on frequency of IJVVI in normal adult population [13,14] and differently to Rabe’s et al. [11] results of IJVVJ frequency in controls. They found IJVVI only in 10% controls. We suppose that methodology of IJVVI assessment, especially expiratory pressure used in controlled Valsalva maneuver, could be responsible for the differences. Another difference between our and Rabe’s study relates to the patients population [11]. The possible advantage of our study is the recruitment of consecutive patients from Headache Outpatient Clinic under follow up. In Rabe’s study patients were recruited using the study questionnaire from the population based German Headache Study on the prevalence of primary headache.

Doepp et al. [2] published results on IJVVI frequency in exertional headache patients. They found IJVVI in 70% of patients compared to 20% of the control group. They observed IJVVI on the dominant venous drainage side. Knappertz et al. [15] speculated on the association between cough headache, incompetence of jugular vein valve and the pain mechanism. Chuang et al. [1] reported internal jugular or vertebral venous regurgitation in benign cough headache. However, population of this study consisted of patients with renal insufficiency on dialysis treatment [1]. Most of them (68%) had central venous thrombosis, so the diagnosis of benign cough headache is controversial. Some authors found a significantly increased frequency of IJVVI in the TGA patients, which may confirm the role of vein drainage disturbances in pathogenesis of TGA [3-5,14]. Migraine and TGA have some clinical similarities: benign course, sudden onset, completely reversible headache and autonomic symptoms [16]. Also cortical spreading depression has been hypothesized to be a trigger in both TGA and migraine. Incompetence of the jugular vein valve during Valsalva maneuver may lead to decreased intracranial venous return and increased intracranial pressure. Increased intracranial pressure might be a potential trigger factor for headache. Because the mechanism of migraine attacks is still unclear, the hypothesis of increased intracranial pressure as a provoking factor for migraine attack and cortical spreading depression seemed to be attractive. However, the results of this study negate that migraine might be associated with jugular vein valve incompetence.

It has to be said that our study possesses some limitations. The most important is methodology and difficulties of IJVVI assessment: it was assumed that the duration of the jet is expression of severity of IJVVI. We defined IJVVI according to Nedelmann [13] as a retrograde flow duration more than 1.2 sec. However, volume of the insufficiency jet may be very different with the same duration, depending on jet width or area at the level of the leaflets. This is however difficult to estimate taking into account the possible eccentric direction of the jet. The other limitation of our study is a small number of the analyzed groups both among the migraine and the control group. A larger population would definitely make our results more reliable and convincing.

In conclusion our results don’t support the hypothesis on possible link between IJVVI and migraine. To our knowledge it is the second report on the lack of association between these two conditions.

## Abbreviations

IJVVI: Internal jugular vein valve incompetence; IHS: International headache society; VM: Valsalva maneuver; MANOVA: multivariable analysis of variance; MO: Migraine without aura; MA: Migraine with aura; TGA: Transient global amnesia.

## Competing interests

The author(s) declare that they have no competing interests.

## Authors’ contributions

All authors read and approved the final manuscript.
